# Report of Cases of Scarlatina, Having Relation to the Question of the Contagiousness of That Diseases

**Published:** 1845-02

**Authors:** J. K. Mitchell

**Affiliations:** Professor of the Practice of Medicine in Jefferson Medical College of Philadelphia


*Report of Cases of Scarlatina, having relation to the question of the contagiousness of that disease.* By J. K. Mitchell, M.D., Professor of the Practice of Medicine in Jefferson Medical College of Philadelphia.—Some years ago I was summoned to attend at the residence of an intimate friend, two of whose children, aged four and six respectively, had been simultaneously attacked by convulsions. On my arrival, they were quiet and drowsy, the spasmodic movements having entirely ceased. These children were, at the time of attack, in different apartments, and while the mother was busy in aiding the elder, who happened to be in her chamber and presence, one of the servants brought the other in her arms from the parlor below. No difference in time or symptoms marked distinctively these cases.

I inferred that either these children had taken some poison at the same time, *per orem*, or had been exposed at the same instant to some noxious *effluvium*, or that they were simultaneously attacked by a contagious disorder. As the patients were soon restored to sense and animation, and complained only of a sense of oppression, and presented much heat of skin, and very rapid pulses, (135 and 142,) I supposed that they were about to have scarlatina; a disease at that time epidemic to a moderate degree.

The most careful inquiry could detect no instance of exposure to a case of that disease. The children had been confined to the house by the inclemency of the wintry weather, and no visitor had been received from any infected quarter; neither was there any case in the immediate neighborhood. These appeared sufficient reasons for a doubt as to the correctness of my suggestion, on the part of the head of the family, who believed in the propagation of scarlatina solely by contagion. But on the following day, *about twenty-four hours* after the attack, the characteristic eruption of scarlet fever put an end to the difficulty as to the nature of the disease. The cases were then attributed to general causes, atmospheric or epidemic; but as no other children, of which there were many in the immediate vicinity, were seized, the question of origination seemed still difficult to perfectly settle.

The cases pursued the usual course, neither of them being as severe as might have been expected from the violence of the onset; but both were, by symptoms and stages and consequences, fully characterized.

After the inquiry as to origin had ceased, the severe illness of the child of a friend of the family, *unheard of before*, led to an apparent solution of the difficulty. It was then remembered that exactly eight days before the attack of the children, a frock had been borrowed from that friend as a pattern; and both the children, being nearly of one size, had tried it on. That frock, being a favorite of the child then ill with the scarlet fever, had been hung on the post of the crib to please it; and had been, when sent for, wrapped up in the sick-room, and thus conveyed, without thinking of contagion, to the house of the borrower, at a distance of nearly two squares.

Three years after the period at which these two cases happened, a commercial gentleman, of high character and beneficent activity, was accosted at the door of his warehouse by a poor woman who carried in her hand a basket of cakes. She stated that her child was dead of scarlet fever, and that she had been compelled to go out in search of the means of making a decent interment. Having had some previous knowledge of the woman, and believing her story therefore to be true, and wishing to aid her, he bought, at a high price, the cakes and the basket, and he and his son, a youth of fifteen years of age, without due consideration, ate some of the cakes. About a week afterwards, on the same day, both were seized with a chilliness followed by fever, sore-throat and scarlet rash, characteristic of scarlatina, and were subsequently very ill. At that time scarlatina prevailed in the district of Southwark, but I did not know of a single case in the city proper. The woman was from Southwark.

The apparent capriciousness of the contagious principle in scarlatina, must have been observed by almost every practitioner. In one instance, the introduction of a case into a family or village has been followed by a general prevalence of the disease in the house or town, while in other and numerous instances it has ceased with a case or two; seeming to languish under apparently the most favorable circumstances. This discrepancy can be explained only by the supposition that, as in certain floral conditions, unfertile germs or cells are sometimes produced, or that there is found in the air, or the physical condition of the recipients, disqualifying potency. The only other probable assumption is that of the existence of two different diseases, so much alike in external phenomena as to forbid discrimination, in the present state of our knowledge.

That the second of these suppositions is sometimes, if not always, the most probable, is sustained by the fact that the virus of *variola* and *vaccinia*, when carefully selected in one place, sometimes fails to propagate disease in another place. Even small-pox, when brought from sea into the Sincapore

Hospital, did not for nearly two years extend to the residents of the house or the place, while, during that period, all the efforts to vaccinate were abortive. So soon, however, as the vaccinators were successful, the small-pox was observed to spread also. The new doctrine of the propagation of contagious diseases by cells or their nuclei, which possess a qualified vitality and power of assimilation and reproduction, leads to the opinion that these germs are sometimes destroyed or disarmed by some other poison.—*Medical Examiner.*

